# Description of two new species of bat fleas of the genus *Araeopsylla* (Siphonaptera) from Kenya and Madagascar with notes on miscellaneous bat fleas

**DOI:** 10.3897/zookeys.572.7823

**Published:** 2016-03-15

**Authors:** Michael W. Hastriter

**Affiliations:** 1 Monte L. Bean Life Science Museum, Brigham Young University, 290 MLBM, P.O. Box 20200, Provo, Utah 84602-0200, U.S.A.

**Keywords:** *Araeopsylla goodmani*, *Araeopsylla smiti*, *Dampfia*, key, *Lagaropsylla*

## Abstract

The flea genus *Araeopsylla* Jordan and Rothschild, 1921 contains nine species distributed throughout the Palaearctic, Ethiopian and Oriental Regions primarily on mollosid bats. A new species of bat flea, *Araeopsylla goodmani*, is described. This new species is represented by three females collected from one male specimen of the mollosid bat *Chaerephon jobimena* Goodman & Cardiff, 2004 from Fianarantsoa Province, Madagascar. A second new species, *Araeopsylla smiti*, is described from one male from the Rift Valley, Kenya. It was collected from the molossid bat *Chaerephon bivittatus* (Heuglin, 1861). This represents the first record of *Araeopsylla* in Kenya. Previous records of *Araeopsylla* in the Malagasy region included *Araeopsylla martialis* (Rothschild, 1903) from Reunion Island and Madagascar. One hundred fifty-eight specimens (64♂, 94♀) of *Araeopsylla martialis* were collected from 67 specimens (flea intensity of 2.4 fleas per host) of *Mormopterus jugularis* (Peters, 1865) across three provinces of Madagascar (Fianarantosa, Toamasina, and Toliara). *Mormopterus jugularis* is clearly a common host for *Araeopsylla martialis*. *Dampfia grahami grahami* (Waterston, 1915) is also reported from *Eptesicus matroka* (Thomas & Schwann, 1905) which is the first record from this host species and the first time the genus *Dampfia* has been documented in Madagascar. Although *Lagaropsylla consularis* Smit, 1957 and *Lagaropsylla idae* Smit, 1957 have been reported in Madagascar previously, *Mops leucostigma* Allen, 1918 is a new host record for *Lagaropsylla idae*. The flea intensity of *Lagaropsylla idae* (64♂, 83♀) on 28 specimens of *Mops leucostigma* was extremely high at 5.3 fleas per host. A key to the genus *Araeopsylla* is provided.

## Introduction

There are currently nine species represented in the flea genus *Araeopsylla* Jordan & Rothschild, 1921 (Lewis 2006) The distribution of *Araeopsylla* is wide-spread, extending across Africa, Madagascar, southern Europe, the Middle East, and Southeast Asia. Members of the genus primarily parasitize bats of the families Emballonuridae and Molossidae. Beaucournu and Fain (1983) and Beaucournu (2004) provided geographical and host lists for Ischnopsyllidae of continental Africa, while Beaucournu and Fontenille (1993) catalogued the fleas of Madagascar. Other papers include specific accounts of miscellaneous small collections of *Araeopsylla*. Although the genus has a broad geographical distribution, specimens are not commonly collected.

During the early 1970’s, the author was associated with a project conducted by the late Hank W. Setzer, Department of Mammalogy, National Museum of Natural History that included the collection of small mammals and their ectoparasites across most of the African countries. The fleas collected were made available to the author and were studied for several years. During those early flea studies, the author recognized a new species of *Araeopsylla* and has maintained the single male specimen for 40+ years in anticipation that the female might be discovered. To date, no additional specimens of this species have been discovered.

The Field Museum of Natural History, Chicago, IL conducted mammal studies in Madagascar and also collected ectoparasites from those mammals. The fleas were provided to the author and they were subsequently identified and returned to the Field Museum. Among the material examined were three female specimens representing a new species of *Araeopsylla* and several new country and host records. These two new *Araeopsylla* taxa from Kenya and Madagascar will be described herein. Additional records of bat fleas from Ghana, Kenya, and Madagascar will also be documented and discussed.

## Methods

Details of the genitalia of the whole mounted specimen of *Araeopsylla smiti* (described below) were difficult to visualize. Therefore the specimen was photographed, dissolved off the microscope slide with xylene, dissected, and remounted in Canada balsam. Images were prepared using an Olympus BX61 Compound Microscope, Olympus CC12 digital camera accompanied with an Olympus Microsuite™ B3SV program. This system was also used to measure fleas in accordance with anatomical markers annotated in Hastriter and Eckerlin (2003). References to “flea intensity” implies the mean number of fleas from hosts that were positive for respective flea species. The primary types of *Araeopsylla goodmani* and *Araeopsylla smiti* were deposited in the Field Museum of Natural History (FMNH), Chicago, IL and the National Museum of Natural History, Washington, D.C., respectively. One paratype of *Araeopsylla goodmani* (SMG-13344-2) was deposited in the FMNH collection and one paratype (SMG-13344-3) in the Brigham Young University flea collection (BYUC).

Madagascar records of bat fleas were extracted from data bases of the FMNH flea collections for which the author provided original species identifications. The additional species annotated herein were all retained in the FMNH flea collection (some mounted on microscope slides and others preserved in alcohol) with exception of those retained in the BYUC noted under “Material Examined” sections. For brevity, collectors are listed as: A. Kofi (AK), B.J. Hayward (BJH), C.B. Robbins (CBR), Fanja H. Ratrimomanarivo (FHR) and (RHF), Steve M. Goodman (SMG).

## Results

### Ischnopsyllidae Ischnopsyllinae

#### Araeopsylla goodmani

Taxon classificationAnimaliaSiphonapteraIschnopsyllidae

Hastriter sp. n.

http://zoobank.org/EF787F18-2E1A-4E8B-9499-A1E98915E53A

Figs 1–5 , 6–7

##### Diagnosis.

Female distinguished from all other *Araeopsylla* species by the shape of the caudal margin of S-VII. The caudal margin lacks lobes but has a broad concave margin extending to near the ventral margin which terminates in a right angled lobe. The apex of the lobe has a small sinus (Fig. 6).

##### Description.

**Figures 1–5.**
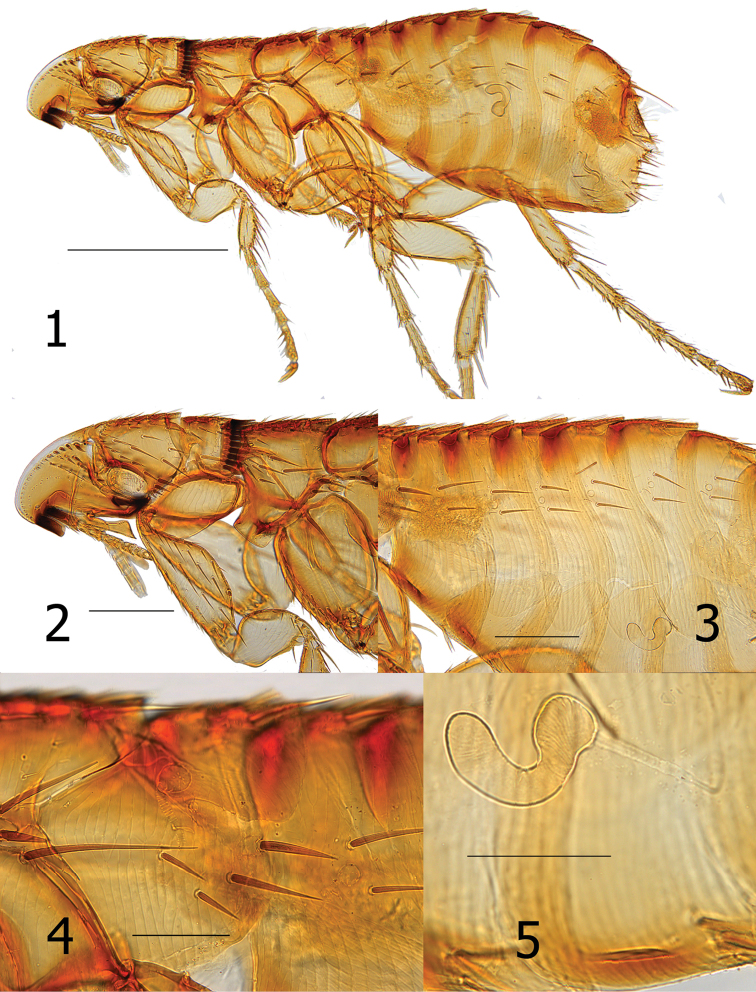
*Araeopsylla goodmani* sp. n., holotype female (SMG-13344-1). **1** Overview of flea **2** Head and pronotum **3** Abdomen (note banding on terga and space between main rows of setae) **4** Metepimeron **5** Spermatheca (note banding on sterna). Scale: 500 µ (**1**); 200 µ (**2–3**); 100 µ (**5–6**).

Head. Frons smoothly rounded; frontal row of 19 setae, each successively stouter from oral angle to top of falx; more dorsal setae spiniform. Area between margin of frons and frontal row of setae clear, white, without surface structure except at extreme upper limit with one placoid pit. Eight minute setae post-frontal row and group of 10 mixed setae (spiniform, short and long) dorsal to row of eight setae. Of these, one seta adjacent to eye, very stout and long, extending beyond posterior margin of head. Gena darkly sclerotized, tapering to upturned apex. Eye vestigial, dark pigmented area merging with gena. Two genal teeth; anterior most tooth broader and blunter than posterior tooth. Pre-oral tuber divided into three portions; most posterior strongly hooked downward to pointed apex. Falx well demarcated. Post-antennal area with row of six stout setulae along dorsal margin of antennal fossa. Four rows of setae dorsal to setulae (4, 2, 3, 8); posterior main row without intercalaries and ventral four spiniform and grouped together (characteristic of the genus). Occiput with three dorsal incrassations. Antennal scape with a few minute setae. Margin of pedicel with hyaline extension over first three segme1nts of clavus; along margin of hyaline area are five or six fine long setae extending to seventh or eighth segment of clavus. Maxilla truncate; shaped like a trumpet at apex. Labial palpus of five segments (excluding bulbous palp-bearing segment); penultimate segment longer than other segments (all being quite short). Labial palpus extending about half length of fore coxa. Length of maxillary palpus similar to labial palpus. Galea and lacinia shorter than labial palpus (Figs 1–2).

Thorax. Pronotum with 18–20 ctenidia; all shorter than length of pronotum. Ctenidia tapered to point, but not sharp at apex. Pronotum with main row of setae minute; more anterior scattered small setae and one long ventral seta. Each thoracic tergum with dorsal incrassations. Twelve stout setae grouped over sclerotic dome of pleural rod. Pleural rod merges with sclerotic dome slightly behind middle of dome. Mesosternum truncate; metasternum diminished but oblique along margin. Metanotum with horizontal row of four setae near interface with dorsal incrassations; one short spinelet at dorsal apex. Lateral metanotal area with one short and one long seta. Metepisternum with one long seta at dorsal margin. Four or five stout (nearly spiniform) setae below level of spiracle on metepimeron. Spiracle on metepimeron large and round (Fig. 4).

Legs. Upper portion of fore coxa very narrow; marginal row of six long setae on upper caudal margin. About 17–18 long lateral setae excluding marginals. All femora lacking lateral or mesal setae. Fore femorotibial joint with one long seta, Meso- and metafemorotibial joints each with two long setae. Lateral surface of fore tibia with six setae; meso- and metatibiae each with eight setae. Dorsal margin of fore tibia with about 10 dorsal notches; meso- (2, 2, 1, 2, 2, 1, 2, 2) and metatibiae (2, 2, 1, 2, 1, 1, 2, 2) each with eight dorsal notches. From proximal to distal, each succeeding tarsus shorter than preceding segment. Each distitarsomere with five lateral plantar bristles; proximal pair placed between second pair on plantar surface (Fig. 1, 4).

Unmodified abdominal segments. Spiracles round on T-II-VII; each segment with one dorsal incrassation and heavily pigmented band extending below incrassation. Terga each with one row of three small setae; single dorsal seta separated from two more ventral setae by large gap. One seta of each row located below spiracle. Heavy sclerotization on ventral surface of S-II–VII. Single row of setae on S-II–VI (1, 2, 2, 3, 3). One antesensilial bristle at margin of T-VII; with internal sclerotized incrassation at base of bristle. Two minute setae on each side of antesensilial bristle (Fig. 3).

**Figures 6–7.**
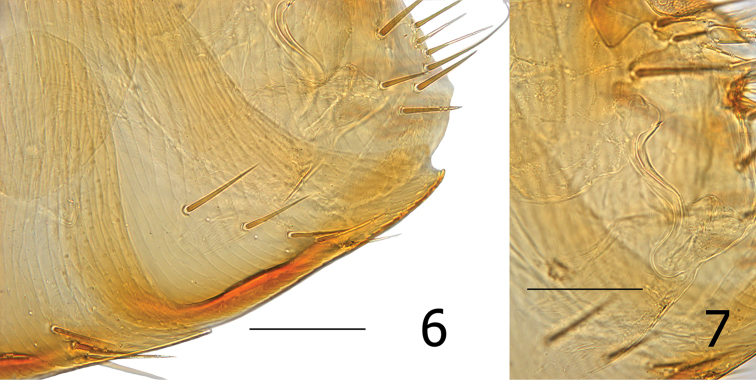
*Araeopsylla goodmani* sp. n., holotype female (SMG-13344-1). **6** Sternum seven **7** Bursa copulatrix. Scale: 100 µ.

Modified abdominal segments. Dorsal portion of T-VIII sclerotized cephalad to trumpet shaped spiracle; all setae below spiracle eight. About 14 setae grouped on apical portion of T-VIII. Caudal margin of S-VII concave to near ventral margin terminating in truncate lobe with small apical sinus. Ventral margin of S-VII with heavy sclerotization; with oblique row of four to six lateral setae (Fig. 6). Sternum eight reduced; without setae. Bursa copulatrix undulate; moderately sclerotized entire length (Fig. 7). Hilla of spermatheca more than twice length of bulga; bulga spherical with cribriform area at ventor. Junction of bulga and hilla hardly distinguishable (Fig. 5).

##### Dimensions.

Female holotype: 2.2 mm, female average: 2.1 mm (n = 3), range: 2.0–2.2 mm.

##### Etymology.

The new species epithet *goodmani* is named in honor of its collector, Dr. Steven M. Goodman, Field Museum of Natural History, Chicago, IL for his untiring efforts and excellent contributions to the field of mammalogy, specifically for his work on bats and small mammals in Madagascar from which these specimens were obtained.

##### Remarks.

Although the respective male and female sexes of the two new flea species described in this paper were both collected from the bat genus *Chaerephon* representing two species [*Chaerephon bivittatus* (Heuglin, 1861) from Kenya and *Chaerephon jobimena* Goodman and Cardiff, 2004 from Madagascar], they do not represent the same species of flea. *Chaerephon bivittatus* and *Chaerephon jobimena* are allopatric in their distributions. Although there exists some sexual dimorphism among fleas, these females differ drastically from the male described below as *Araeopsylla smiti*. Characteristics examined included major differences in the nature of the genal teeth, pre-oral tuber, pronotal comb, shape of the gena, variations in chaetotaxy of head and abdomen, and abdominal incrassations.

*Araeopsylla lumareti* Smit, 1958 (known only from the male sex) could potentially represent the male of this new species for which only females are known; however, this is doubtful based on their differences in hosts, morphology, and geographical remoteness. *Araeopsylla lumareti* is known only from the type locality in Cambodia from “bat guano” opposed to the occurrence of *Araeopsylla goodmani* in Madagascar from *Chaerephon jobimena*, which is endemic to Madagascar. The frontal row of setae of males of *Araeopsylla lumareti* are comprised of “small setae” and the occiput is “without marked dorsal incrassations” (Smit 1958). The frontal row setation of *Araeopsylla goodmani* range from small setae to spiniform setae, the occiput is with marked dorsal incrassations, and the first genal tooth is much broader than that of *Araeopsylla lumareti*. Based on these observations, I am confident that the male of *Araeopsylla lumareti* does not represent the male counter-part of *Araeopsylla goodmani*. Additional collecting of fleas from *Lagaropsylla jobimena* in Madagascar and from the temple of Angkor-Vat in Cambodia is needed to discover the males of *Araeopsylla goodmani* and the females of *Araeopsylla lumareti*.

Several species of the bat genus *Chaerephon* have yielded several bat flea species of the genus *Lagaropsylla* (Beaucournu 2004, Beaucournu and Fain 1983, Beaucournu and Fontenille 1993, Beaucournu and Kock 1994a, and Klein and Uilenberg 1966), but this is the first record of *Araeopsylla* collected from the bat genus *Chaerephon* throughout Madagascar or tropical Africa.

##### Type material examined.

**Madagascar, Fianarantsoa Province**: Isalo, 3.8 km NW Ranohira, along Namaza River (22°32'24"S, 45°22'48"E), *Chaerephon jobimena* ♂, 1 XII 2002, SMG, (SGM-13344-1, holotype ♀, SGM-13344-2, paratype ♀, FMNH) (SGM-13344-3, paratype ♀, BYUC).

#### Araeopsylla smiti

Taxon classificationAnimaliaSiphonapteraIschnopsyllidae

Hastriter sp. n.

http://zoobank.org/28BA81B1-6B62-4770-9A14-92E4F176A188

Figs 8–13

##### Diagnosis.

Distinguished from all other species of *Araeopsylla* by the details of the telomere and distal arm of S-IX. The telomere is acutely pointed at apex and has a broadly rounded lobe along its ventral margin (Fig. 10). The ventral lobe at the base of the distal arm of S-IX that is present in other *Araeopsylla* species is short, pencil-like and without an expanded lobe at its apex. The ventral lobe of the new species is drastically longer and adorned with an ornate apical lobe (Fig. 11). The eighth sternum, unlike all other species, has a lobe (paired) with tufts of long, coarse setae (Fig. 11).

**Figures 8–13.**
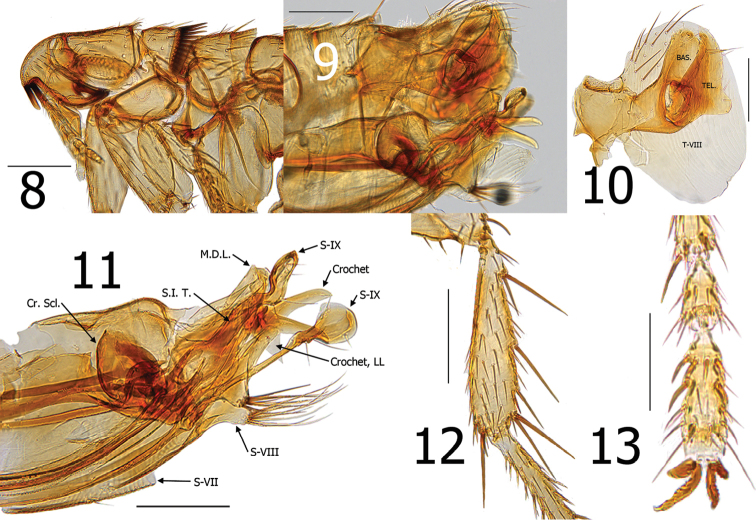
*Araeopsylla smiti* sp. n., holotype male (BJH-5634). **8** Head and pronotum **9** Terminal segments, before dissection **10** Basimere, telomere, and eighth tergum **11** Aedeagus, eighth sternum, and distal arm of ninth sternum, (Cr. Scl. = Crescent Sclerite, LL = Lower Lobe, M.D.L. = Median Dorsal Lobe, S.I.T. = Sclerotized Inner Tube) **12** Hind tibia **13** Fourth and fifth segments of distitarsomere 2. Scale: 200 µ (**8–12**), 100 µ (**13**).

##### Description.

Head. Margin of frons gradually thickened from falx to oral angle. Frontal row of 9–10 minute setae. Area between frons and frontal row of setae with small punctate structures and two placoid pits. Area between frons and frontal row not white or clear, but moderately sclerotized. One placoid pit postad to frontal row of setae near base of genal tooth. Seven to eight minute setae postad to frontal row; group of 6–7 variable sized setae near ventral margin of antennal fossa. Eye fused into darkly sclerotized genal lobe. Genal lobe tapered and broadly rounded at apex; with minute apical tooth. Setae in occipital area rather randomly arranged. Five setulae along dorsal margin of antennal fossa; each as long as other randomly arranged setae in occipital area. Two small setae postad to antennal fossa in position of what is usually 4-5 spiniform setae in other *Araeopsylla* species. Apex of scape enlarged; three long setae along upper margin and three marginal setae at apex. Pedicel with several fine setae along apical margin; none extending beyond first segment of antenna. Clavus asymmetrical; not extending beyond margin of head. Five segmented labial palpus extended to 1/3 length of fore coxa. Proximal segment of five segmented labial palpus rather bulbous in form (Fig. 8).

Thorax. Pronotum with 26 ctenidia; each acutely pointed and only slightly shorter than length of pronotum. Setae on pronotum randomly arranged. Two dorsal and two ventral pseudosetae under mesonotal collar. Metanotum with three rows of setae; two marginal short, stout spinelets at dorsal apex of sclerite. Pleural rod nearly centrally attached to sclerotic dome. Ventral portion of metasternum lobed downward between coxae. Pleural arch absent. Metepisternum with squamulum and one long seta at dorsal margin. Metepimeron with nine setae; spiracle large and round.

Legs. Fore coxa with ~40 lateral setae. Fore femur with 2 minute setae on mesal surface; none on lateral surface. Mesal surface of mesotibia with single row of five setae; multiple setae on lateral surface. Mesal surface of metatibia with single row of seven setae; multiple setae on lateral surface. Meso- and metatibiae with five well defined notches; two setae at each. Multiple single setae interspersed along margins between defined notches. Each tarsal segment longer than adjacent more distal segments on meso- and metatarsi. Five lateral plantar bristles on all distitarsomeres; first pair displaces between second pair. Two pre-apical plantar bristles on each distitarsomere (Figs 12–13).

Unmodified abdominal segments. Single spinelet on apex of T-I. Globular sclerotized incrassation at base of each terga (T-I–VII). Pigmented banding extends slightly ventrally from each incrassation. Each terga with single uninterrupted row of setae; one seta below level of each round spiracle. Single long antesensilial bristle. Ventral margin of each sternite is heavily sclerotized. Sternites II–III with one minute seta in row; S-IV–VI with two minute setae in each row.

Modified abdominal segments. Basimere without lobes or sinuses. Telomere with large lobe on lower ventral margin; a few fine setae along ventral margin of telomere above lobe. Tergum VIII encompassing basimere and telomere with all setae restricted to dorsal half of sclerite (Fig. 10). Sternum VIII with large lobe bearing tuft of long coarse setae. Crescent sclerite of aedeagus long, thin, and inverted so tectum is directed cephalad. Sclerotized inner tube straight, somewhat broad. Crochet with prominent truncate upper lobe and sharp ventral lobe. Distal arm of S-IX with sigmoid lobe similar to many bat species, but what is usually a small pencil-like lobe in other species, this lobe is greatly extended and expanded at its apex. The expansion bears two small setae at base of expansion, two setae on apico-dorsal surface, and one seta on ventral margin of expansion. A group of very fine hairs adorns the base of the ventral lobe of the distal arm of S-IX (Figs 9–11).

##### Etymology.

Mr. F.G.A.M. Smit, during his long tenure at the British Museum, London was without doubt, a major contributor to our knowledge of the global flea fauna. It is thus fitting to name this flea *smiti* in his honor as a noun in apposition.

##### Remarks.

This is the first record of the genus *Araeopsylla* occurring in Kenya, although the genus has been recorded throughout tropical Africa.

##### Type material.

Holotype ♂, Kenya, Rift Valley, Maji Moto, 4.8 km W of Lake Harrington (00°16'00"S, 03°6'04 “E), *Chaerephon bivittatus* (USNM Host 437-287), 25 VIII 1968, BJH-5634.

#### Araeopsylla martialis

Taxon classificationAnimaliaSiphonapteraIschnopsyllidae

(Rothschild, 1903)

Ceratophyllus martialis Rothschild, 1903
Ischnopsyllus martialis Rothschild, 1906: 187–188
Araeopsylla martialis Jordan & Rothschild, 1921: 146; Hopkins and Rothschild 1956: 323–325; Lumaret 1962: 12; Klein and Uilenberg 1966: 53; Beaucournu and Fain 1983: 458, 460, 465; Beaucournu and Fontenille 1993: 79; Lewis 2006: 44: Marcus 1961: 190.

##### Remarks.

Although *Araeopsylla* is generally collected only in very small numbers across its range, *Araeopsylla martialis* is exceptional. Sixty-seven individual *Mormopterus jugularis* specimens yielded one or more specimens of *Araeopsylla martialis*. These were collected in three provinces of Madagascar (Fianarantosa, Toamasina, and Toliara). A total of 158 (64♂, 94♀) specimens were harvested from the 67 specimens of *Mormopterus jugularis*, yielding a flea intensity of 2.4. One male specimen of *Araeopsylla martialis* was collected from *Rousettus madagascarensis*. Other species of bats did not harbor *Araeopsylla martialis*. Its occurrence on a *Rousettus* sp. is likely an accidental association.

##### Material examined

**(BYUC). Madagascar, Fianarantsoa Province**: Fianarantsoa église FLM (21°27'32"S, 47°04'36"E), 1190 m, *Mormopterus jugularis* ♂, 18 XI 2004, FHR, 1♂, 1♀.

#### Dampfia grahami grahami

Taxon classificationAnimaliaSiphonapteraIschnopsyllidae

(Waterston, 1915)

Ischnopsyllus grahami Waterston, 1915: 115; Bedford 1932: 462.
Dampfia grahami grahami Smit 1954: 148–149; Smit 1955: 215–216; Hopkins and Rothschild 1956: 312; Marcus 1961: 184–187; Beaucournu 2004: 205.

##### Remarks.

A total of 15 specimens (8♂, 7♀) was collected from four specimens of the Malagasy endemic *Neoromicia matroka* (Thomas & Schwann, 1905). This flea is not commonly collected and the only known existing records include the holotype ♂ from *Eptesicus capensis* = *Neoromicia capensis* (A. Smith, 1829) from Cape Town, South Africa, 3♀ specimens from Natal, and 2♀ from Orange Free State, South Africa (bat host species undetermined). *Neoromicia capensis* is widely spread across sub-Saharan Africa. Although not found in Madagascar, Goodman et al. (2012) considered *Neoromicia capensis* to be the closest ally and sister group to *Neoromicia matroka*. The current specimens represent a new country and host record and substantially increase the known number of specimens available for study. The hosts for *Dampfia* are thus far restricted to the family Vespertilionidae.

##### Material examined

**(BYUC). Madagascar, Toamasina Province**: Andasibe, Ankazinina (18°56'38"S, 48°24'46"E), 970 m, *Eptesicus matroka* (Thomas & Schwann, 1905) (RHF-58), 18 IX 2004, FHR, 1♀.

#### Lagaropsylla consularis

Taxon classificationAnimaliaSiphonapteraIschnopsyllidae

Smit, 1957

Lagaropsylla consularis Smit, 1957: 167, 1958: 242; Marcus 1961: 201; Smit 1968: 13; Hubbard 1969: 55; Ribeiro 1974: 143; Beaucournu and Fain 1983: 454–455; Beaucournu and Guiguen 1991: 129; Beaucournu and Fontenille 1993: 80; Beaucournu and Kock 1994b: 199, 1996: 164; Beaucournu 2004: 206; Lewis 2006: 48.

##### Remarks.

*Lagaropsylla consularis* has been reported previously in Madagascar and is among the more common species in the genus, primarily parasitizing *Chaerephon pumilus*. *Chaerephon pumilus* has a broad range from Yemen to Senegal, south to South Africa and Madagascar. *Neoromicia somalicus* (Thomas, 1901) from which *Lagaropsylla consularis* was collected in Kenya, is also found in Madagascar. Reported primarily on molossid bats, *Lagaropsylla consularis* has also been documented on hipposiderid and vespertilionid bats (Beaucournu 2004 and Smit 1957).

##### Material examined

**(BYUC). Kenya**: Rift Valley, Maji Moto, *Neoromicia somalicus* (USNM-436733), 23 XIII 1968, BJH-5592, 2♂, 2♀. **Madagascar, Toamasina Province**: Beforona, Bureau de Poste (18°53'21"S, 48°34'39"E), 560 m, *Chaerephon pumilus* ♂ (RHF-84), 21 IX 2004, FHR, 1♂, 1♀; same data except Anjiro village (18°53'40.31"S, 47°58'24.06"E), *Chaerephon pumilis* ♂ (RHF-502), 5 II 2005, 1♂, 1♀.

#### Lagaropsylla hoogstraali

Taxon classificationAnimaliaSiphonapteraIschnopsyllidae

Smit, 1957

Lagaropsylla hoogstraali Smit, 1957: 171–172, 1964: 44.
Lagaropsylla traubi Klein, 1967: 127–131; Smit and Wright 1978: 41; Lewis and Lewis 1990: 156. (Synonym).
Lagaropsylla hoogstraali Ribeiro, 1974: 144; Beaucournu and Fain 1983: 455; Beaucournu and Fontenille 1993: 79–80; Beaucournu 2004: 207; Lewis 2006: 48.

##### Remarks.

*Lagaropsylla hoogstraali* has been documented in Angola, Rwanda, Sudan, Zaire and Madagascar. Although there are few collections of this flea, most have been collected from *Mops midas* (Sundevall, 1843), a broadly distributed bat in continental Africa and Madagascar. Ratrimomanarivo et al. (2007) concluded that the subspecific populations of *Mops midas* in continental Africa and Madagascar were invalid. The flea *Lagaropsylla hoogstraali* occurring on *Mops midas* in both regions would support the conclusions of Ratrimomanarivo et al. (2007).

##### Material examined

**(BYUC). Madagascar, Mahajanga Province**: Ambondromamy, Cite de la Gendarmerie (16°26'03"S, 47°09'26"E), 50 m, *Mops midas miarensis* = *Mops midas* ♀ (RHF-823), 13 III 2005, FHR, 1♂; same data except *Mops midas* ♂ (RHF-824), 1♂; and *Mops midas* ♂ (RHF-825), 1♀. **Toliara Province**: Sakaraha, EPP (22°54'26"S, 44°31'48"E). 480 m, *Mops midas* ♂ (RHF-262), 20 X 2004, FHR, 1♀.

#### Lagaropsylla idae

Taxon classificationAnimaliaSiphonapteraIschnopsyllidae

Smit, 1957

Lagaropsylla idae Smit, 1957: 165–167; Marcus 1961: 196–199; Smit 1964: 44; 1968: 13–14.
Lagaropsylla setzeri Segerman, 1970: 3–5; Smit and Wright 1978: 43; Lewis and Lewis 1990: 139. (Synonym)
Lagaropsylla idae Ribiero, 1974: 144; Beaucournu and Fain 1983: 455; Beaucournu and Guiguen 1991: 129; Beaucournu and Kock 1994b: 199, 1996: 164; Beaucournu 2004: 207; Lewis 2006: 48.

##### Remarks.

Beaucournu (2004) suggested that *Mops condylurus* (A. Smith, 1833) is the principle host of *Lagaropsylla idae*. Specimens of *Lagaropsylla idae* in my collection from Ghana and Kenya were also collected from *Mops condylurus*. This host is widely distributed across Africa but is not found in Madagascar. *Mops leucostigma*, endemic to Madagascar, is very closely allied to the mainland species *Mops condylurus*. A total of 147 specimens (64♂, 83♀) to include those listed in the “Materials examined” section below and those preserved in alcohol in the FMNH was collected from 28 specimens of *Mops leucostigma*, yielding a flea intensity of 5.3 fleas per host. An average of 5+ fleas per host is a very high flea intensity for any bat flea, as bat fleas in general, are usually found in extremely low numbers. *Mops leucostigma* is the preferred host of *Lagaropsylla idae* in Madagascar and it was not collected from any other bat species in Madagascar.

##### Material examined

**(BYUC). Ghana, Eastern Region**: Teshi, Accra Plains (05°34"N, 00°00'6"W), *Mops condylurus* (USNM-412535), 26 XI 1967, CBR-1931, 2♂; Volta, Denu (06°06’N, 00°10'9"E), *Mops condylurus* (USNM-412667), 31 VIII 1967, AK-138, 1♂, 1♀. **Kenya, Eastern Region**: Mtoto Andei (02°41’S, 03°8'08"E), *Chaerephon pumilus* (Cretzschmar, 1830) (USNM-437019), 9 VII 1968, BJH-4846, 1♂; same data except *Mops condylurus* (USNM-437225), BJH-4844, 1♀; Kiboko (02°12’S, 03°7'42"E), *Mops condylurus* (USNM-437181), 4 VII 1968, BJH-4684, 1♂. **Kenya, Rift Valley**: Maji Moto, *Nycticeinops schlieffeni* (Peters, 1859) (USNM-436774), 24 VIII 1968, BJH-5610, 1♂, 2♀. **Madagascar, Toamasina Province**: Andasibe, Bureau du Poste (18°55'18"S, 48°25'18"E), 950 m, *Mops leucostigman* ♂ G.M. Allen, 1918 (RHF-13), 14 IX 2004, FHR, 1♀; Anjiro, Andranoalina (18°52'57"S, 47°58'15"E), 850 m, *Mops leucostigma* ♂ (RHF-532), 7 II 2005, FHR, 1♂. **Toliara Province**: Sakaraha, Bureau Eau et Forets (22°54'34"S, 44°31'20"E), 470 m, *Mops leucostigma* ♂ (RHF-202), 15 X 2004, FHR, 1♀, same data except *Mops leucostigma* ♀ (RHF-206), 1♂; Andranovory, Hòpital (23°08'37"S, 44°08'41"E), 500 m, *Mops leucostigma* ♂ (RHF-179), 13 X 2004, FHR, 1♂, same data except *Mops leucostigma* ♂ (RHF-181), 1♀.

### Key to the species of *Araeopsylla*

**Table:** 

1	Males (*Araeopsylla goodmani* sp. n., male unknown)	**2**
1’	Females (*Araeopsylla lumareti* and *Araeopsylla smiti* sp. n., females unknown)	**11**
2(1)	Acetabular bristles arranged on prominent long lobe of basimere (lobe longer than wide)	**3**
2’	Acetabular bristles along margin of basimere, not borne on lobe	**6**
3(2)	Ventral margin of telomere with a sinus and subtending lobe	**4**
3’	Ventral margin without a sinus or lobe	**5**
4(3)	Caudal margin of T-VIII truncate. Lobe on caudal margin of telomere rounded, not hooked downward (Cambodia)	***phnomensis***
4’	Caudal margin of T-VIII narrowing to rounded lobe. Lobe on caudal margin of telomere hooked downward (Cambodia)	***immanis***
5(3’)	Ventral margin of telomere convex; dorsal margin concave (Cambodia)	***lumareti***
5’	Ventral and dorsal margins nearly straight (Thailand)	***elbeli***
6(2’)	Apex of manubrium spatulate (Rwanda)	***faini***
6’	Apex of manubrium not spatulate	**7**
7(6’)	Basal lobe of distal arm of S-IX long and modified. Telomere acutely pointed at apex. Truncate lobe at apex of S-VIII with tuft of long, coarse setae (Kenya)	***smiti* sp. n.**
7’	Basal lobe of distal arm of S-IX short, without leaf-like apical lobe. Telomere rounded and blunt at apex. Sternum VIII without lobe bearing tuft of long setae	**8**
8(7’)	Apex of manubrium sharp and turned downward. Sinus present above acetabular bristles on basimere (Kenya, Angola, Lesotho, South Africa)	***scitula***
8’	Manubrium and basimere otherwise	**9**
9(8’)	Crochet without hook-like lobes (Italy)	***gestroi***
9’	Crochet with hook-like lobes	**10**
10(9’)	Basimere quadrate on dorso-apical margin. Telomere broadens towards apex and extends beyond apex of basimere (Réunion Island, Madagascar)	***martialis***
10’	Basimere rounded on dorso-apical margin. Telomere somewhat parallel sided, rounded at apex, and sub equal in length to basimere (Egypt)	***wassifi***
11(1’)	Hilla of spermatheca hardly longer than bulga (Thailand)	***elbeli***
11’	Hilla distinctly longer than length of bulga	**12**
12(11’)	Caudal margin of S-VII concave to margin of terminal truncate ventral lobe; ventral lobe with small sinus at apex (Madagascar)	***goodmani* sp. n.**
12’	Caudal margin of S-VII straight (not concave), or with lobes	**13**
13(12’)	Bursa copulatrix rather straight; without sigmoid-like curves	**14**
13’	Bursa copulatrix not straight, but with various sigmoid-like curves	**15**
14(12’)	Caudal margin of T-VIII slightly convex with vertical row of three short spiniform setae near apical margin of convexity. Spiracle VIII broadened at apex (Egypt)	***scitula***
14’	Caudal margin of T-VIII more straight; without row of three spiniform setae. Spiracle VIII rounded at apex (Egypt)	***wassifi***
15(13’)	One closely arranged vertical row of six spiniform setae at caudal margin of T-VIII (Réunion Island, Madagascar)	***martialis***
15’	Chaetotaxy of caudal margin of T-VIII otherwise	**16**
16(16’)	Caudal margin of S-VII without lobe (Cambodia)	***phnomensis***
16’	Caudal margin of S-VII with lobe	**17**
17(16’)	Apex of ventral margin of S-VII extends beyond dorsal lobe (Cambodia)	***immanis***
17’	Dorsal lobe projects beyond apex of ventral margin	**18**
18(17’)	Dorsal lobe on caudal margin of S-VII broad, subtended by a broad shallow sinus (Italy)	***gestroi***
18’	Broad angular lobe on margin of S-VII without subtending sinus (Rwanda)	***fain***

## Supplementary Material

XML Treatment for Araeopsylla goodmani

XML Treatment for Araeopsylla smiti

XML Treatment for Araeopsylla martialis

XML Treatment for Dampfia grahami grahami

XML Treatment for Lagaropsylla consularis

XML Treatment for Lagaropsylla hoogstraali

XML Treatment for Lagaropsylla idae
